# A 3D printed mimetic composite for the treatment of growth plate injuries in a rabbit model

**DOI:** 10.1038/s41536-022-00256-1

**Published:** 2022-10-19

**Authors:** Yangyi Yu, Kristine M. Fischenich, Sarah A. Schoonraad, Shane Weatherford, Asais Camila Uzcategui, Kevin Eckstein, Archish Muralidharan, Victor Crespo-Cuevas, Francisco Rodriguez-Fontan, Jason P. Killgore, Guangheng Li, Robert R. McLeod, Nancy Hadley Miller, Virginia L. Ferguson, Stephanie J. Bryant, Karin A. Payne

**Affiliations:** 1 Colorado Program for Musculoskeletal Research, Department of Orthopedics, University of Colorado Anschutz Medical Campus, Aurora, CO USA; 2 Shenzhen Key Laboratory of Musculoskeletal Tissue Reconstruction and Function Restoration, Department of Bone and Joint Surgery, Shenzhen People’s Hospital (The Second Clinical Medical College Jinan University, The First Affiliated Hospital, Southern University of Science and Technology), Shenzhen, China; 3 Department of Mechanical Engineering, University of Colorado Boulder, Boulder, CO USA; 4 Materials Science and Engineering Program, University of Colorado Boulder, Boulder, CO USA; 5 Applied Chemicals and Materials Division (647), National Institute of Standards and Technology (NIST), Boulder, CO USA; 6 Department of Electrical, Computer and Energy Engineering, University of Colorado Boulder, Boulder, CO USA; 7 Musculoskeletal Research Center, Children’s Hospital Colorado, Aurora, CO USA; 8 BioFrontiers Institute, University of Colorado Boulder, Boulder, CO USA; 9 Department of Chemical and Biological Engineering, University of Colorado Boulder, Boulder, CO USA; 10 Gates Center for Regenerative Medicine, University of Colorado Anschutz Medical Campus, Aurora, CO USA

**Keywords:** Implants, Regenerative medicine

## Abstract

Growth plate injuries affecting the pediatric population may cause unwanted bony repair tissue that leads to abnormal bone elongation. Clinical treatment involves bony bar resection and implantation of an interpositional material, but success is limited and the bony bar often reforms. No treatment attempts to regenerate the growth plate cartilage. Herein we develop a 3D printed growth plate mimetic composite as a potential regenerative medicine approach with the goal of preventing limb length discrepancies and inducing cartilage regeneration. A poly(ethylene glycol)-based resin was used with digital light processing to 3D print a mechanical support structure infilled with a soft cartilage-mimetic hydrogel containing chondrogenic cues. Our biomimetic composite has similar mechanical properties to native rabbit growth plate and induced chondrogenic differentiation of rabbit mesenchymal stromal cells in vitro. We evaluated its efficacy as a regenerative interpositional material applied after bony bar resection in a rabbit model of growth plate injury. Radiographic imaging was used to monitor limb length and tibial plateau angle, microcomputed tomography assessed bone morphology, and histology characterized the repair tissue that formed. Our 3D printed growth plate mimetic composite resulted in improved tibial lengthening compared to an untreated control, cartilage-mimetic hydrogel only condition, and a fat graft. However, in vivo the 3D printed growth plate mimetic composite did not show cartilage regeneration within the construct histologically. Nevertheless, this study demonstrates the feasibility of a 3D printed biomimetic composite to improve limb lengthening, a key functional outcome, supporting its further investigation as a treatment for growth plate injuries.

## Introduction

**Fig. 1:**
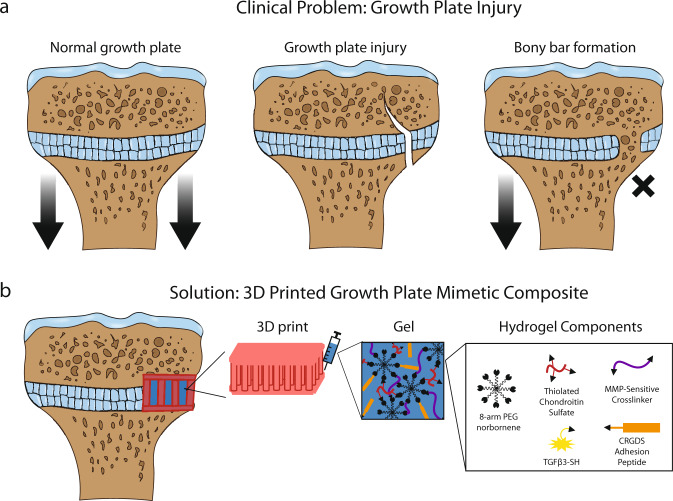
Overview of the clinical problem of growth plate injury and the use of a 3D printed growth plate mimetic composite as a solution. **a** Schematic of uniform bone elongation in a normal proximal tibial growth plate (left), which can be disrupted by a growth plate injury (center), and lead to partial or complete growth arrest due to bony bar formation (right). **b** Schematic of the implanted 3D printed growth plate mimetic composite (gel + 3D print) after resection of the bony bar. The composite contains a 3D print infilled with the MMP-sensitive, cartilage-mimetic, PEG-based hydrogel (Gel). The hydrogel components are shown on the right.

The growth plate (or physis) is a cartilaginous structure that lies between the epiphysis and metaphysis of children’s long bones. It is a dynamic tissue that drives bone elongation through the process of endochondral ossification^1^. Normal bone elongation is uniform, with the growth plate becoming progressively thinner until complete closure occurs at skeletal maturation^2^. However, the growth plate also represents a weak point of the developing pediatric skeleton due to the juxtaposition of relatively soft cartilage against hard bone. Injuries involving the growth plate make up ~15–30% of all childhood skeletal injuries^3,4^ and of these up to 30% can lead to lifelong orthopedic problems^5^. The main concern with growth plate injuries is the formation of unwanted bony repair tissue known as a “bony bar” that leads to bony tethering of the epiphyseal and metaphyseal bones (Fig. 1a). This bony bar restricts local growth and can result in angular deformities or limb length discrepancies.

Current clinical treatment options include resection of the bony bar when it occupies <33% of the growth plate and implantation of an interpositional material to avoid reformation of bony tethering and to prevent subsequent growth deformities^6^. On the other hand, when the bony bar is larger, more invasive procedures are warranted (i.e., corrective osteotomies or bone transport)^7^. To date the most common interpositional material is autologous fat^8–12^, but studies have shown poor to fair outcomes in 16–63% of patients treated with this approach^9–11,13^. Current interpositional materials are designed to act simply as a spacer, and thus are limited in that they do not function to replace the native growth plate tissue or in themselves induce growth through the normal biological process of endochondral ossification. If the desired outcome is a fully restored growth plate and new growth at the former site of bony tethering, a tissue engineering and regenerative approach is needed.

The growth plate is subject to a complex local mechanical and molecular environment. Dynamic compressive forces have been shown to stimulate epiphyseal cartilage growth, while shear and hydrostatic pressures more likely drive cartilage ossification^14,15^. Studies have also shown that overloading can suppress longitudinal bone deposition^16–18^. Thus, a successful tissue-engineered construct would require a structure that is stiff enough to support loading and prevent collapse but also that fosters an environment for stem cell proliferation and differentiation. Previously explored scaffold chemistries for growth plate tissue engineering include chitin^19,20^, agarose^21,22^, atelocollagen^23^, poly(lactic-co-glycolic acid) (PLGA)^12,24^, and demineralized bone matrix^25^. The primary purpose of these scaffolds was delivery of biologically active components (e.g., stem cells, cytokines, etc.) at the injury site, rather than provision of robust structural support. For example, PLGA was used to create a porous, degradable scaffold that locally delivered insulin-like growth factor 1, in a rabbit growth plate injury model^24^. Although these structures resulted in promising indications for growth factor stimulation of chondrogenesis, the scaffold itself was found to fragment during the final stages of degradation in vitro. The development of a treatment approach capable of providing both relevant biologically active cues to support regeneration and robust structural support, would be an important next step toward developing a clinically relevant treatment approach.

Three-dimensional (3D) printing provides a feasible method to fabricate structures that meet the mechanical needs of the biological system, combined with a structural design (low volume fraction) that allows for the inclusion of a bioactive niche^26^ capable of supporting the regeneration of growth plate cartilage. Limitations of many printing methods include resolution and the need to print extra material to support overhangs. Stereolithographic digital light processing (DLP) is one promising printing method that addresses these shortcomings. DLP printing involves rapid liquid-solid transition under light irradiation and thus resolution is a function of the projector, making resolutions <50 µm possible^27^. Furthermore, DLP printing does not require a support material for overhanging features which allows for more complex designs compared to other 3D printing techniques.

The objective for this study was to develop a 3D printed growth plate mimetic composite for use following a bony bar resection to prevent bony bar reformation, support cartilage regeneration, and restore limb growth. To do so, we first characterized the mechanical properties of rabbit growth plate to guide the creation of the 3D printed growth plate mimetic composite. A poly(ethylene glycol) (PEG)-based resin was used in DLP 3D printing to create a stiff 3D printed structure made of vertical pillars (Fig. 1b). This simple structural design was selected to provide vertical axial support. The lattice structure was used to connect the vertical pillars and was designed with minimum thickness and in a grid pattern with open regions rather than a solid layer to allow for easier infiltration of local cells. We then infilled the stiff structure with a soft cartilage-mimetic hydrogel comprised of crosslinked PEG as the base chemistry and to which extracellular matrix analogs of chondroitin sulfate (ChS) and cell adhesive peptides, RGD, and the chondrogenic growth factor, transforming growth factor β3 (TGFβ3), were tethered (Fig. 1b). The instructive hydrogel was designed to be degradable through matrix metalloproteinase (MMP)-sensitive crosslinks, which was confirmed to support cell viability and promote cartilage growth^28–31^. We investigated the ability of our mimetic composite (gel + 3D print) to induce chondrogenic differentiation of rabbit mesenchymal stromal cells (rMSCs) in vitro. We then evaluated its efficacy as a regenerative interpositional material in a rabbit model of growth plate injury and compared it to an untreated control, cartilage-mimetic hydrogel only, and a fat graft as the clinical gold standard. Limb growth, tibial angle, morphological bone changes, and histological analysis of the repair tissue between the different treatment groups were performed. A second in vivo study compared different iterations of our growth plate mimetic composite with altered structural properties. This study combined 3D printing and a bioactive hydrogel to develop a mimetic composite as a potential treatment of growth plate injuries that addresses both the need for mechanical support and cartilage regeneration following a bony bar removal.

## Results

### Native growth plate tissue mechanics guide properties of tunable 3D printed growth plate mimetic composite

**Fig. 2:**
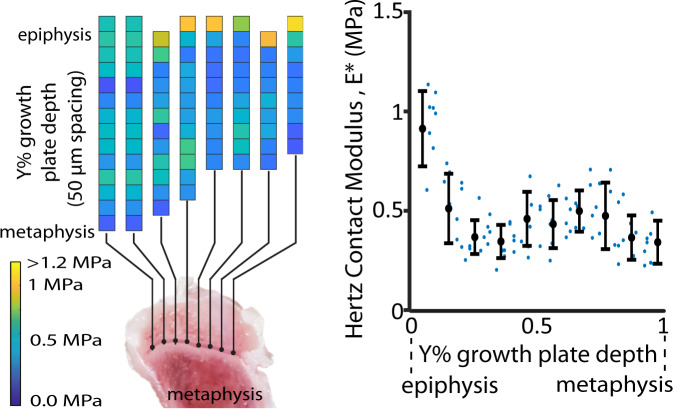
Mechanical characterization of a New Zealand white rabbit growth plate tissue. Modulus results were obtained from micro-indents in arrays spanning the growth plate cartilage at eight different locations (left). Hertz contact moduli were averaged anterior–posterior and evaluated as a function of depth across the growth plate (i.e., Y% with respect to the epiphyseal and metaphyseal junction) with a bin size of 10%. Small blue dots represent individual data points, bold black dots represent mean ± standard deviation (error bars).

To guide the creation of a 3D printed structure for the growth plate mimetic composite (gel + 3D print) (Fig. 1b), native rabbit growth plate tissue was characterized by micro-indents spanning the epiphyseal bone, growth plate cartilage, and metaphyseal bone. After removing indents crossing over into bone, Hertz contact modulus of the growth plate cartilage ranged 0.2–1.14 MPa with variability across the depth of the tissue, but with a mean value of 0.48 MPa (Fig. 2).

**Fig. 3:**
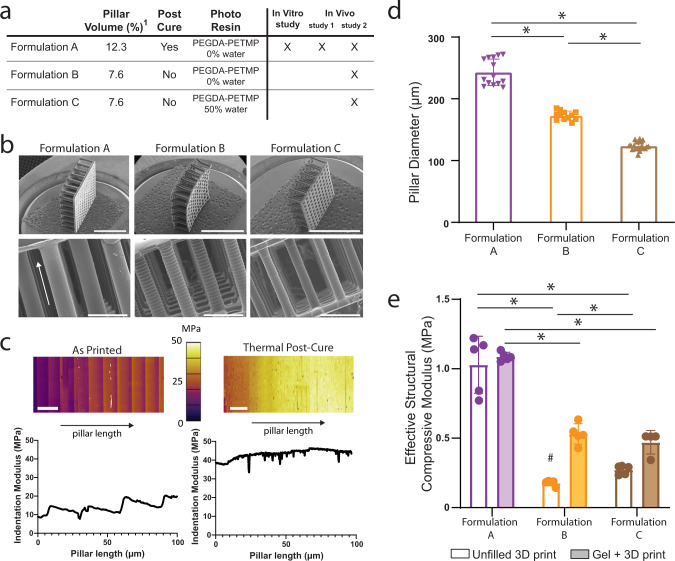
Characterization of the different gel + 3D print formulations used in this study. **a** Table showing the pillar volume (^1^pillar volume % is based on CAD design), post-cure conditions, and photo resin used for the 3D print for each formulation. **b** SEM images of all three scaffold designs. Scale bar on top row of images is 3 mm and higher-resolution images (bottom row) scale bar represents 500 μm. (arrow represents pillar length). **c** 3D printed structure evaluated via AFM showing fluctuations in mechanical properties down the length of the pillar due to printing process (left, as printed) and the improved mechanical properties and reduction in variance after thermal post-cure process (right, thermal post-cure). Scale bars represent 20 µm. **d** Pillar diameter measured via SEM showing all three formulations have significantly different pillar diameters once printed. * denotes significant differences (*p* < 0.05; Kruskal–Wallis test). **e** Effective structural compressive modulus of the unfilled 3D print and gel + 3D print composite using Formulation A, Formulation B, and Formulation C (unfilled 3D print represented with empty fill bars, Gel + 3D print represented with solid fill bars). Results show Formulation A was significantly different than Formulation B and C, but no differences in compressive modulus were seen between Formulations B and C when filled with the cartilage mimetic hydrogel. *denotes significant differences (*p* < 0.05; one-way ANOVA, Tukey’s HSD post hoc) between formulations, and # denotes significant difference (*p* < 0.05; *t*-test) between the filled and unfilled 3D print. Data represent mean ± standard deviation (error bars).

The modulus data were used to guide the creation of three formulations of the 3D printed structure (Formulation A, B, C) with modifications to the volume fraction of printed pillars, application of a thermal post-cure, and addition of a solvent (i.e., water) in the resin formulation (Fig. 3a). Representative scanning electron microscopy (SEM) images show the fidelity of the vertical pillar array in the 3D printed structure (Fig. 3b). The DLP 3D printing process causes depth-dependent mechanical properties within each print layer, which was observed by atomic force microscopy (AFM) and resulted in a mean indentation modulus value of 19.4 ± 5.1 MPa (Fig. 3c, left). Thermal post-curing eliminated the depth-dependent effects in each layer, stiffening the material by 2.2-fold to a mean indentation modulus value of 43.2 ± 0.5 MPa (Fig. 3c, right). This post-cure led to the significant reduction of the characteristic saw-tooth feature of DLP printed parts, which is more evident in the SEM images of Formulation B and C which did not undergo the post-cure (Fig. 3b). While initial CAD designs used identical pillar diameters of 200 µm, the variations in the formulation led to differences in the pillar diameters, as measured from SEM images (Fig. 3d). The thermal post-cure treatment in Formulation A resulted in pillars measuring 243 ± 21 μm in diameter. Without the thermal post-cure (Formulation B), the pillar diameter decreased 29% and with the addition of a solvent in the resin (Formulation C) the pillar diameter decreased further by 28%. The compressive modulus of the base cartilage-mimetic hydrogel has been previously measured and reported to be 54 ± 10 kPa^32^. The resin material used to 3D print the structure had an elastic compressive modulus of 32 ± 2 MPa. When unfilled, the 3D print using Formulation A, B, and C had effective structural compressive modulus of 1.03 ± 0.21 MPa, 0.17 ± 0.03 MPa, and 0.27 ± 0.03 MPa, respectively (Fig. 3e). Once infilled with the cartilage-mimetic hydrogel, the gel + 3D print Formulation A had a similar effective structural compressive modulus of 1.09 ± 0.03 MPa (Fig. 3e). However, once infilled, the gel + 3D print Formulation B and C had improved effective compressive moduli of 0.53 ± 0.07 and 0.47 ± 0.07 MPa, respectively (Fig. 3e), suggesting that the cartilage mimetic hydrogel may help support the pillars and prevent buckling, allowing them to support higher loads.

### 3D printed growth plate mimetic composite supports chondrogenesis of rMSCs

**Fig. 4:**
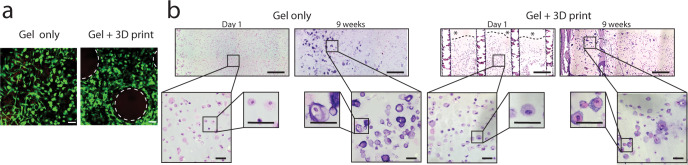
The 3D printed structure supports rabbit MSC viability and morphology in the cartilage-mimetic hydrogel. **a** Representative live/dead staining of samples at 9 weeks (scale bar = 50 µm). Scaffold pillars in the Gel + 3D print condition are outlined in dashed white line. **b** Representative H&E images of the plastic embedded Gel only and Gel + 3D print conditions at day 1 and at 9 weeks. 10x images (scale bar = 500 µm) highlight the distribution of cells in each condition. * denotes small areas where lack of observable cells is a result of sectioning artifacts, dashed line shows where section was torn. 40x insets (scale bar = 50 µm) highlight round cell morphology and deposition of pericellular matrix in both conditions after 9 weeks of culture.

To assess whether the presence of a stiff 3D printed structure affects rMSC viability and morphology, cells were encapsulated in the cartilage-mimetic hydrogel, and were either directly polymerized (gel only) or used to infill a 3D printed structure and then polymerized (gel + 3D print). Samples from both conditions were stained with calcein AM (live cells) and ethidium homodimer (dead cells) to assess cell viability after nine weeks of culture (Fig. 4a). Minimal evidence of dead cells (red) and abundant presence of live cells (green) indicate that the cells survived the nine weeks of culture. Distribution of green staining throughout constructs in both conditions suggest that the encapsulated cells remained dispersed throughout the hydrogels over the culture period. Representative hematoxylin and eosin (H&E) microscopy images of both the gel only and gel + 3D print conditions (Fig. 4b) confirmed a relatively uniform distribution of cells across conditions at day one. Higher-resolution images demonstrate that the encapsulated rMSCs had a similar round morphology within the cartilage-mimetic hydrogel in both conditions. After 9 weeks, cells maintained uniform distribution throughout both conditions and displayed a round chondrogenic phenotype, with some deposition of pericellular matrix (PCM) in both conditions. These observations indicate that the 3D structure retains the round cell morphology within the soft mimetic hydrogel.

**Fig. 5:**
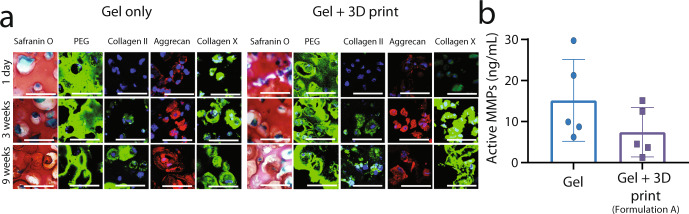
3D printed growth plate mimetic composite supports MSC chondrogenesis. **a** Representative images from histological and immunohistochemical staining from paraffin-embedded samples for both the Gel only and Gel + 3D Print conditions after 1 day, 3 weeks, and 9 weeks of culture (scale bar = 50 µm). Due to difficulty in sectioning, the images for the gel + 3D print are at a closer magnification than the gel only condition. Images include staining for PEG to assess hydrogel degradation, sGAGs (Safranin O/fast green), collagen II, and aggrecan to assess chondrogenic differentiation of encapsulated cells, and collagen X to assess hypertrophy. **b** Total matrix metalloproteinases (MMPs) secreted by the cells in the media over the course of the 9-week study. Data represent mean ± standard deviation (error bars).

To assess the chondrogenic differentiation of the rMSCs in the gel only or gel + 3D print conditions, Safranin-O/Fast Green staining and immunofluorescence for aggrecan and collagen types II and X were performed after one day, three weeks, and nine weeks of culture (Fig. 5a). Safranin-O/Fast Green staining indicated that sGAGs were present throughout the study period across both conditions. Positive staining at 1 day was expected, as the hydrogel formulation includes ChS. There was positive staining at 3 and 9 weeks in regions around the cells, which were devoid of polymer (as highlighted by the absence of PEG staining). This increase in sGAGs in the PCM indicates that the encapsulated cells synthesized sGAGs. Moreover, newly synthesized sGAGs contribute to the positive staining within the hydrogel as aggrecan monomers can diffuse through a crosslinked hydrogel. The cellular regions in the 3D printed structure showed similar sGAG staining to the gel only. Positive PEG staining after nine weeks in both conditions indicated that the soft hydrogel had not completely degraded, but no qualitative differences in degradation of the hydrogel were seen across the two conditions. Additionally, both conditions showed an absence of collagen II and aggrecan at day 1 and a progressive increase in their expression through week 9. Collagen X protein expression, a marker of hypertrophy, was observed at day 1 for both conditions and increased in expression over the study period. Overall, these results demonstrate that the rMSCs underwent chondrogenesis within the gel only and gel + 3D print conditions with the only chondrogenic inducing growth factor tethered into the hydrogel and absent in the culture medium. Analysis of the total MMPs in the media revealed that cells in both conditions produced MMPs and although the gel + 3D print condition had a lower average amount, the differences were not statistically different (Fig. 5b). Taken together, these results demonstrate that the 3D printed growth plate mimetic composite supports chondrogenesis of encapsulated rMSCs, resulting in a neocartilage ECM composed of collagens II and X and aggrecan. These findings indicate that the 3D structure maintains the ability of the cartilage-mimetic hydrogel to induce chondrogenesis of the encapsulated rMSCs.

### 3D printed growth plate mimetic composite promotes increased bone elongation

**Fig. 6:**
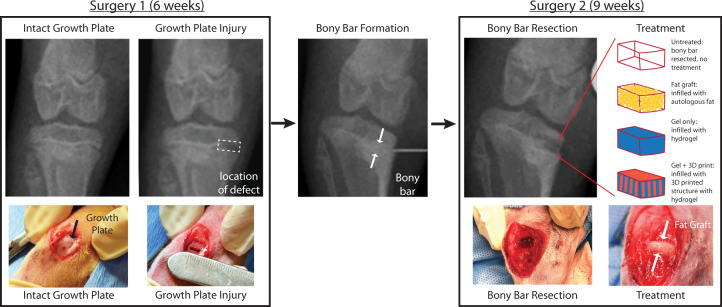
Overview of rabbit growth plate injury model using a two-surgery approach. The first surgery to create an initial growth plate injury occurs at 6 weeks of age, where a 1 mm drill bit attached to a Dremel is used to make a 5 × 5 × 1 mm defect. A bony bar forms in the injured area during the 3 weeks after surgery. The second surgery when the rabbit is 9 weeks of age uses a 2 mm drill bit to remove the bony bar and reopen the defect in a 6 × 6 × 2 mm region. After bony bar resection, one of four treatment options are applied: untreated, fat graft, gel only, gel + 3D print.

To evaluate the efficacy of the 3D printed growth plate mimetic composite in vivo, we used a rabbit growth plate injury model and a two-surgery procedure^33^. During the first surgical procedure, when the rabbit was 6-weeks old, a 5 mm × 5 mm × 1 mm area of the proximal anterior-medial tibia growth plate was removed from the right limb (Fig. 6, Surgery 1). The left limb remained intact and served as an uninjured control. Three weeks after the initial surgery, bony repair tissue formed which tethered the epiphyseal and metaphyseal bone. In addition to the bony bar, the injured limb was shorter than the uninjured limb with a mean limb length difference of 2 mm and had a tibial angle difference of 6°. During a second surgery, the bony bar was resected as would be done clinically, and one of four treatments was implemented: (1) untreated, (2) autologous fat graft as the clinical gold standard, (3) cartilage-mimetic hydrogel (gel only), and (4) 3D printed growth plate mimetic composite (gel + 3D print, Formulation A) (Fig. 6, Surgery 2).

**Fig. 7:**
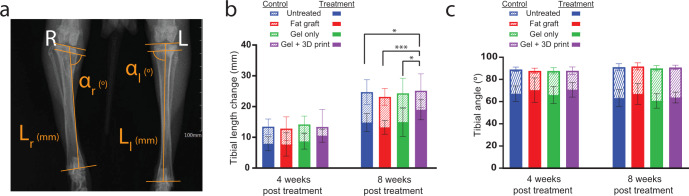
3D printed growth plate mimetic composite promotes increased bone elongation. **a** Representative radiograph showing the method for measuring tibial length (L) and tibial angle (α). **b** Tibial length change for the control limbs (dashed) and treatment limbs (solid bars) at 4- and 8-weeks post treatment. While there was no difference in tibial length change amongst the control limbs, the gel + 3D print condition had significantly more tibial growth compared to other treatments. Data represent mean ± standard deviation (error bars). *Denotes significant difference in tibial length change of treatment limbs (*p < 0.05, ***p < 0.001; two-way ANOVA, Tukey’s HSD post hoc). **c** Tibial angle of control limbs (dashed) was similar, as were the tibial angles of the treatment limbs (solid bars) across groups. Data represent mean ± standard deviation (error bars).

Tibial length and tibial angle were measured from anteroposterior radiographs taken at the time of second surgery and at 4- and 8-weeks post treatment (Fig. 7a). We evaluated the tibial length change post treatment in both limbs as a measure of bone elongation (Fig. 7b, uninjured control limbs dashed, injured limbs solid bars). The uninjured control limbs had greater lengthening than the treated limbs (*p* < 0.001; two-way ANOVA, Tukey’s HSD post hoc), with no differences observed between control limbs for the different groups (*p* = 0.87; two-way ANOVA, Tukey’s HSD post hoc) (Fig. 7b). Limb length difference between the uninjured (left) and injured (right) limbs was 7.5 ± 3.1 mm and 11.4 ± 3.1 mm for 4- and 8-weeks post treatment, respectively. However, tibial length change across the treatment groups indicated that the gel + 3D print condition had significantly more growth at 8 weeks post treatment compared to all other treatment options (untreated: *p* = 0.02, fat graft: *p* < 0.001, gel only: *p* = 0.02; two-way ANOVA, Tukey’s HSD post hoc) (Fig. 7b). The tibial angle was not significantly different across treatment groups in either the control (*p* = 0.57; two-way ANOVA, Tukey’s HSD post hoc) or treatment limbs (*p* = 0.41; two-way ANOVA, Tukey’s HSD post hoc) (Fig. 7c). At 8 weeks post treatment the mean tibial angle across all control limbs was 91 ± 3°, while the treatment limbs had a mean tibial angle of 63 ± 7°.

**Fig. 8:**
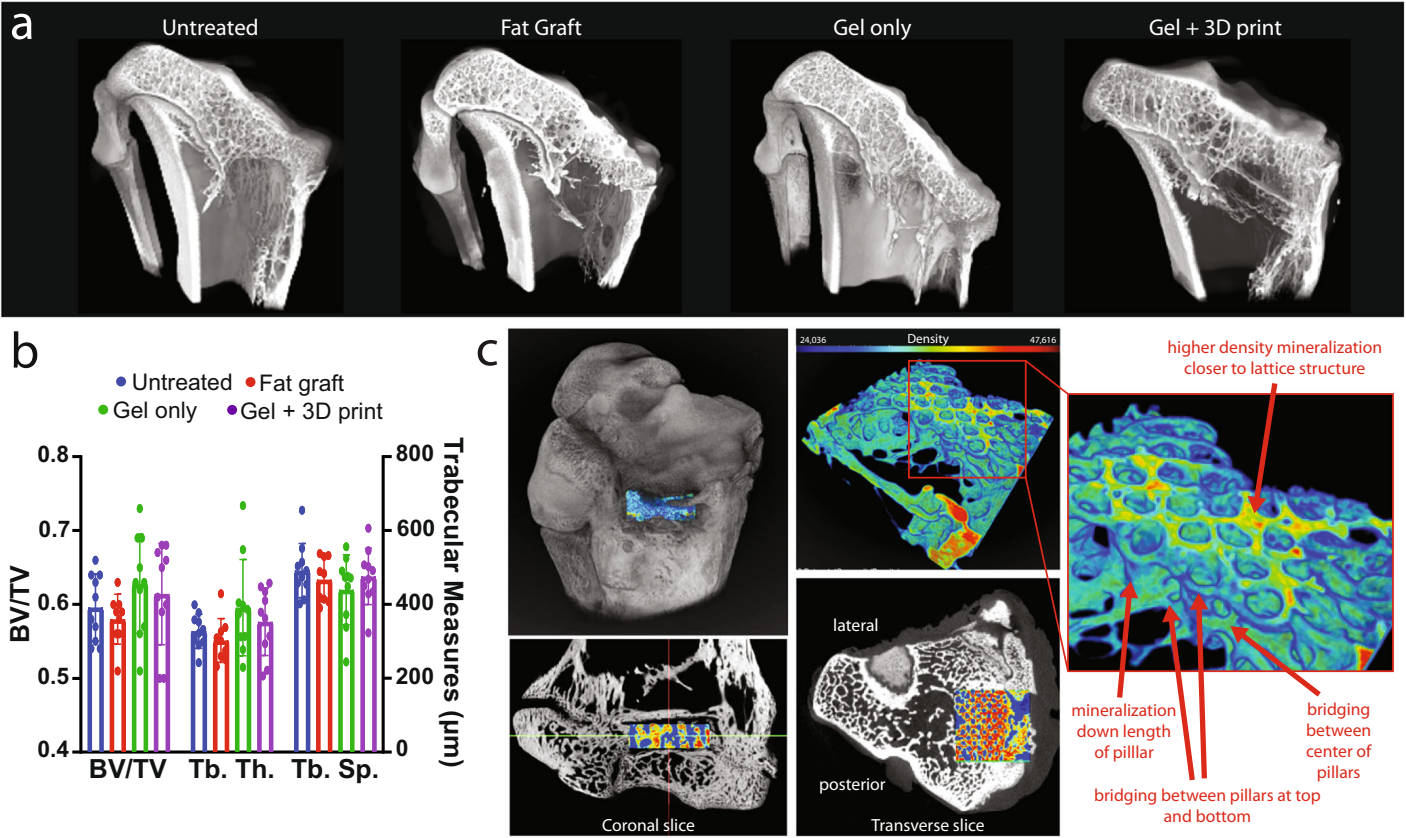
MicroCT assessment at 8 weeks post treatment. **a** Representative microCT images of all treatments clipped at mid-plane to expose a cross-section of the injury site. **b** Bone analysis of all treatment limbs highlighting lack of significant differences across any measure between treatments. Data represent mean ± standard deviation (error bars). **c** Representative Gel + 3D print specimen with the composite region highlighted with a LUT based on density (scaffold attenuation will appear black in gray scale or dark blue in LUT). Panels show 3D view, coronal slice, transverse slice, and ROI of the gel+3D print (cropped to show interior of scaffold excluding lattice). Inset highlights mineralization patterns within the scaffold including higher mineralization towards the lattice structure (top and bottom). There was also mineralization down the length of the pillars but bridging between pillars was less common.

Injured limbs were assessed by microCT at 8-weeks post treatment, and all showed a disrupted growth plate at the injury site (Fig. 8a). No significant differences were observed for BV/TV, Tb.Th, or Tb.Sp (Fig. 8b). BV/TV across all treatment limbs was 0.60 ± 0.01, while Tb. Th. and Tb. Sp. were 339.2 ± 90.3 μm and 471.2 ± 77.2 μm, respectively. Reconstruction of the microCT data and segmentation around the structure suggests that calcified tissue formed around and between the pillars within the gel + 3D print (Fig. 8c).

### Cartilage-mimetic hydrogel and 3D printed growth plate mimetic composite led to limited cartilage-like tissue formation

**Fig. 9:**
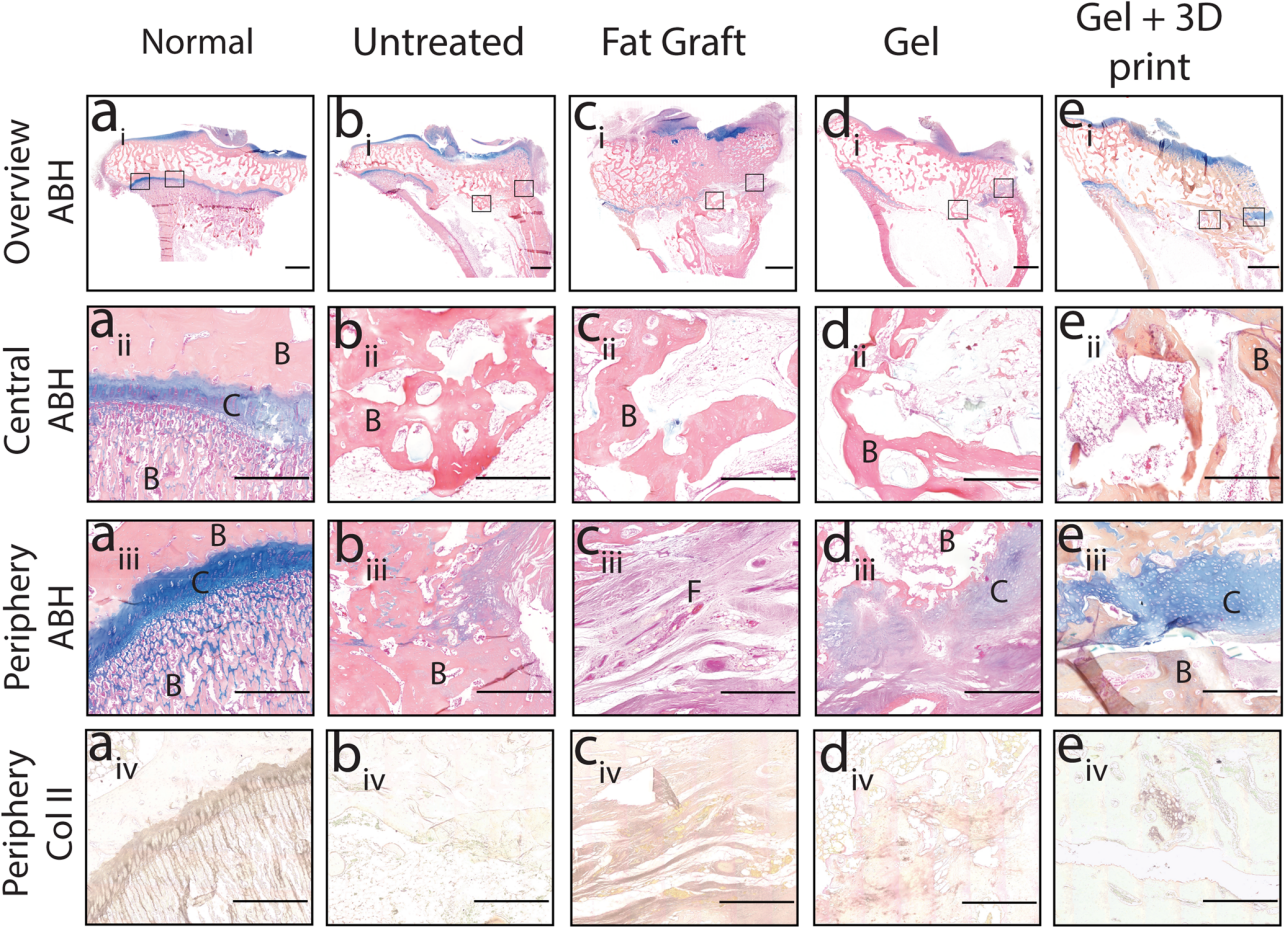
Treatment with the 3D printed growth plate mimetic composite showed peripheral cartilage-like tissue in vivo. Representative Alcian Blue Hematoxylin staining (i–iii) and Collagen type II staining (iv) of the proximal tibia growth plate of **a** an uninjured (Normal) or **b** untreated rabbit, as well as growth plates treated with **c** a fat graft, **d** gel only, or **e** Gel + 3D print. (i) overview ABH images (scale bar = 2000 μm) with the boxed areas shown as a higher magnification image (scale bar = 500 μm) in (ii) the central region of the growth plate and (iii) the periphery. C = cartilage, F = fibrous tissue and B = bone. (iv) Collagen type II immunohistochemistry at higher magnification (scale bar = 500 μm) near the periphery.

To assess the repair tissue that formed at the site of injury, Alcian Blue-Hematoxylin, Eosin Y, and Orange G (ABH) staining was performed. The uninjured limbs had a continuous cartilaginous (blue) growth plate region (Fig. 9a), while the untreated limbs showed mostly bony repair tissue, with a small area of Alcian Blue positive staining at the periphery of the injured area (Fig. 9b). The stained tissue appeared disorganized, with few cells exhibiting a chondrocyte-like morphology. The fat graft treatment had bone in the central region and fibrous tissue in the periphery of the defect area (Fig. 9c). The gel only and gel + 3D print treatments resulted in some bone centrally, but also had peripheral areas with cartilage-like tissue (Fig. 9d, e). In the case of the gel only specimens, the cartilage-like tissue was dispersed in pockets with disorganized chondrocytes. The gel + 3D print samples had mineralized tissue localized adjacent to the pillars, but cartilage-like tissue was seen in the periphery of the injury, particularly above the lattice of the structure. Three representative samples from each treatment group were evaluated semi-quantitatively to determine the percent of the injury which was positively stained with Alcian Blue. The injury ROI was outlined, and a combination of color splitting and thresholding were implemented. The mean percent positive Alcian Blue staining for the untreated, fat graft, gel only, and gel + 3D print conditions were 2.3 ± 0.3%, 2.5 ± 2.5%, 3.7 ± 1.8%, and 4.5 ± 2.8%, respectively. Adjacent tissue sections were also stained for collagen type II (Fig. 9aiv-eiv). While most of the repair tissue in all treatment groups did not stain positive for collagen type II, a small area of positive staining was found proximal to the lattice structure in the Gel + 3D print group.

### Varying the 3D printed structural properties did not significantly change outcomes

**Fig. 10:**
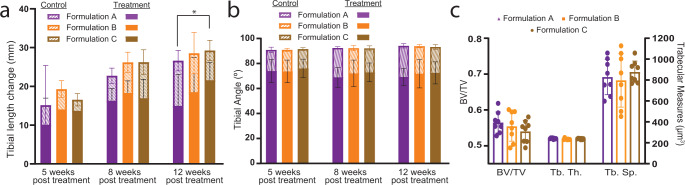
Varying the structural properties of the 3D printed composite does not affect tibial length, angle, or bone metrics. **a** Tibial length change for the control limbs (dashed) and treatment limbs (solid bars) at 5-, 8-, and 12-weeks post treatment using Formulation A, B, or C. A significant difference was observed between Formulation A and C at 12-weeks post treatment in the treatment limb. *Denotes significant difference in tibial length change of treatment limbs (*p < 0.05; two-way ANOVA, Tukey’s HSD post hoc). **b** Tibial angle of control limbs (dashed) was similar, as were the tibial angles of the treatment limbs (solid bars) across groups at both 5-, 8-, and 12-weeks post treatment. **c** No bone metrics measured by microCT were different across scaffold formulations. Data represent mean ± standard deviation (error bars).

To evaluate if 3D printed structural properties (pillar volume ratio, pillar diameter, and effective compressive modulus) played a role in our treatment, we again used our rabbit growth plate injury model (Fig. 6) and evaluated three gel + 3D print formulations (Formulation A, B, and C). Tibial length change was reduced compared to control limbs in all treatment limbs regardless of formulation. However, at 12-weeks post treatment Formulation C, with the lowest overall compressive modulus, had significantly greater tibial growth compared to Formulation A (*p* = 0.047; two-way ANOVA, Tukey’s HSD post hoc) (Fig. 10a). Tibial angle in all injured limbs was significantly different from control limbs, but no differences were seen across the three gel + 3D print formulations (*p* = 0.77; two-way ANOVA, Tukey’s HSD post hoc) (Fig. 10b). There was a significant effect (*p* < 0.002; two-way ANOVA, Tukey’s HSD post hoc) of time with respect to limb length growth. The average tibial length difference between the control and treatment limb increased from 4.5 ± 3.4 mm to 8.0 ± 4.4 mm and 10.0 ± 4.3 mm from 5-weeks to 8-weeks and finally 12-weeks post treatment. The mean tibial angle of the treatment limbs was 75 ± 8°, 71 ± 9°, and 71 ± 9° for 5-, 8-, and 12-weeks post treatment, respectively. No significant differences were observed between any bone metrics across the three different formulations (Fig. 10c).

## Discussion

Injuries to the growth plate that result in bony bar formation can lead to significant limb length discrepancies and angular deformities, which can subsequently cause gait disturbances, low back pain, cosmetic deformity, and early-onset osteoarthritis. Current treatments are directed at removing the bone tethering and preventing its recurrence but do not attempt to regenerate the damaged growth plate. Herein, we utilized 3D printing technology to create a growth plate mimetic composite that meets the mechanical needs of the biological system and contains a bioactive niche capable of supporting chondrogenesis. We characterized the mechanical properties of the rabbit growth plate to instruct the mechanical properties of the stiff 3D printed structure that was then infilled with the cartilage-mimetic hydrogel. In vitro we demonstrated that our growth plate mimetic composite induced chondrogenic differentiation of rMSCs. We observed that degradation of the PEG-based hydrogel provided space for local deposition of aggrecan, collagen II, and collagen X indicating new ECM was being produced within our cartilage-mimetic hydrogel both in gel only and gel + 3D print conditions. In vivo, the 3D printed growth plate mimetic composite allowed for significantly more limb growth compared to other treatment options, but limited cartilage-like tissue was observed, and it was not located within the construct. We also demonstrated that the material and structural properties of our composite material are tunable.

Lacking literature-based values at the time of this work, it was necessary to characterize the native tissue to aid in the design of our composite. Our testing methods determined a Hertz contact modulus which represents an aggregate tissue response and does not account for potential anisotropy. We observed differences in Hertz contact modulus with depth which is in agreement with growth plate studies in other species^34–37^. These modulus values, 0.2–1.14 MPa, were used to guide the creation of three tunable composites with compressive moduli, 0.4–1.12 MPa, encompassing that of native tissue. While only one animal was evaluated in this study, subsequent work on a larger sample size (*n* = 15 animals) has since been published and values are in agreement with those presented here^38^. The open 3D printed structure allows for ease of infilling with a softer and cell instructive hydrogel. Our biomimetic composite is an improvement to other approaches that have been explored in growth plate applications that utilize either a soft hydrogel only (e.g., agarose, collagen, and hyaluronan), which have compressive modulus in the 0.001–0.150 MPa range^21,23,39^ far less than the mean native tissue modulus value of 0.48 MPa found in this study, or that have more physiologically relevant mechanical properties (e.g., PLGA) but lack bioactivity.

In vitro, rMSCs encapsulated in both the gel only and gel + 3D print conditions were well dispersed. There was positive staining for newly synthesized aggrecan, collagen II, and collagen X confirming both chondrogenesis and new cartilage-like ECM production independent of the presence of the 3D structure. The encapsulated rMSCs secreted MMPs, but at levels that did not lead to rapid degradation of the cartilage-mimetic hydrogel, allowing for local ECM deposition and growth. While additional quantitative measures for assessing ECM production including gene and biochemical levels should be considered in future studies, the current data suggest that our composite design allows for independent control over the properties with the incorporation of a tunable stiff 3D printed structure and provides an inductive environment for chondrogenesis afforded by the soft cellular niche.

Impaired bone elongation is a major clinical concern with growth plate injury and bony bar formation. Using a rabbit model of growth plate injury that develops a bony bar and limb length discrepancy, we were able to demonstrate that resection of the bony bar and treatment with a 3D printed growth plate mimetic composite (gel + 3D print) allowed for significantly more limb growth within 8-weeks post treatment compared to all other treatments. This increased bone elongation may be a result of the 3D print alone acting as an interpositional material limiting bony tethering and providing improved mechanical support compared to the other treatments. Thus, a major limitation of the current study is the absence of an empty scaffold condition. Since limb lengthening was improved with the gel + 3D print compared to the gel only condition it is clear the 3D printed scaffold is beneficial, but we are unable to discern the exact mechanism of in vivo efficacy. Additional work is needed to determine the role of the bioactive hydrogel in promoting cartilage regeneration in vivo both independently and as an infill in the 3D printed scaffold.

While the untreated group had bone across most of the defect region, and the fat graft condition displayed bony tissue along with pockets of fibrous tissue particularly along the periphery of the defect, the gel only and gel + 3D print treatments had bony infill in the center of the defect but also had cartilage-like tissue with presence of sGAGs at the periphery. In the gel only condition, cartilage-like tissue was limited to the medial and peripheral most aspect of the treatment site and contained few chondrocyte-like cells. The 3D printed growth plate mimetic composite led to more intense staining for sGAGs with cells showing distinct chondrocyte morphology and some cells staining positive for collagen II. Given that the cartilage-rich regions were not within the 3D print but rather near its periphery and at the proximal edge of the injury area, it is possible that the gel only and gel + 3D printed growth plate mimetic may preserve surrounding growth plate cartilage. However, due to the extensive injury that occurred during the second surgical procedure that extended proximal and distal from the growth plate, as well as the organization of the tissue, it is also possible that these cartilage-like areas, particularly those seen in the gel + 3D print, are neo-tissue formation. Since histology was performed at the end of the 8-week study, it is unknown whether a more robust cartilage tissue formation occurred early on and underwent endochondral bone formation to support bone lengthening. Future studies with earlier time points and an unfilled 3D print could help elucidate the origin of the cartilaginous tissue and the mechanism that led to greater bone elongation with the 3D printed growth plate mimetic composite.

A goal of interpositional materials is to prevent bony bar reformation, however, as reported in other tissue-engineered composite and gel studies^21,24,25^, we observed some degree of bony infill in all treatments. Since microCT was performed at euthanasia and partial growth plate closure had occurred, it is difficult to ascertain if the mineralization in the defect region was normal bone closure, or bony bar reformation. Previous studies have pointed to most interpositional materials being only acutely effective in preventing bony bar reformation. A study by Jin et al., found that their autologous tissue-engineered composite only prevented bony reformation in the first two weeks^25^. Based on in vitro analysis we did not anticipate the extent of mineralization observed in our gel only or gel + 3D prints in the in vivo studies. Future studies might consider evaluating earlier time points to determine the rate of mineralization within the scaffold. Additionally, design modifications to the scaffold including the lattice structure (i.e., altering open space dimensions) might also prevent ingrowth of calcified tissues. We also noted minimal trabecula occupying the space distal to the growth plate. Given that we did not perform microCT on the contralateral control limbs in this study, we are unable to attribute that bone loss to normal age-related processes or a result of our injury model. However, we did not observe differences in the degree of metaphyseal bone reduction across groups, so we do not believe this is related to any treatment modality.

Angular deformity is also a concern with growth plate injuries as it can lead to growth disturbances. In this study, tibial angle of the injured limbs continued to decline despite any of the treatments evaluated. This is likely due, in part, to the timing of the bony resection and subsequent treatments. On average, in the three weeks between the original injury and bony bar formation, when resection and treatment occurred, the tibial angle of the injured limbs changed by 9 ± 5°. While natural correction of angular deformities of up to 35° have been reported in humans^11^, the threshold for corrective osteotomy usually varies from 10 to 35° and is partially dependent on the location and extent of growth plate damage^6^. Moreover, it is not yet clear if the rabbit growth plate injury model is capable of a natural correction. Since we did not perform an osteotomy, the angular deformity present at the time of treatment remained. Experimental growth plate injury and treatment models have varied in the time treatment is delivered. Studies that treated immediately following injury have reported a higher instance of significant reductions in angular deformities and leg length discrepancies^19,39–42^ compared to those that treated 3 weeks post injury^12,21,43^. In our current model, we attempted to simulate a more clinically relevant condition where the injury is treated after presentation rather than at time of injury, but earlier intervention might result in more significant reduction in deformities long term due to the rapid rate of growth in rabbits at this age. Despite this challenge in the rabbit model, our gel + 3D print composite displayed positive effects in limb lengthening and warrants further investigation, even if a follow-up osteotomy may be necessary.

Minimal differences in measured outcomes were observed between the three formulations for the 3D printed structures in the second in vivo study. When comparing the 8-week post treatment time point in the initial study to that of the second, all three formulations had increased tibial growth and reduced tibial angle changes compared to the untreated condition. This finding suggests that a 3D printed structure with a range of pillar diameters and composite moduli from 0.4 to 1 MPa does not affect the positive outcome. Thus, the benefits observed from our 3D printed growth plate mimetic composite are seen across a range of structural properties and appear to result in improved outcomes compared to softer treatments such as fat grafts or soft hydrogels.

In conclusion, we have demonstrated the ability to 3D print a growth plate mimetic composite that led to increased tibial lengthening in a rabbit model of growth plate injury. The composite ultimately did not result in a robust layer of regenerated cartilage-like tissue 8 weeks post treatment. It also remains unclear if the improved tibial lengthening is mainly a result of the additional mechanical support provided by the scaffold or if the scaffold and cartilage mimetic hydrogel infill had a synergistic effect. In addition to improved limb lengthening, the mimetic composite has the advantage of being an off-the-shelf product, while the current clinical gold standard fat graft treatment requires a second surgical incision thus increasing patient risk for infection and recovery time. Further design modifications are necessary to improve in vivo chondrogenesis and reduce mineralized infill, but the results from this study are promising for moving towards using a regenerative interpositional material to treat growth plate injuries.

## Methods

### Native growth plate mechanical characterization

A single tibia sourced from a 9-week-old New Zealand white rabbit (Charles River Laboratories Inc., Wilmington, MA, USA) was used to assess the mechanical properties of native growth plate cartilage. Sections were taken from the sagittal plane of the proximal tibial growth plate and polished to 2500 grit (precision polisher; EXAKT 400CS). Arrays (17×1) of micro-indents spanning the epiphyseal bone, growth plate cartilage, and metaphyseal bone were performed (Hysitron TI 950 TriboIndenter, xZ-500 extended displacement stage, parallel-plate capacitor) at eight evenly spaced locations spanning the sagittal slice. For each array, indents were made with a diamond R = 50 μm spherical probe and spaced 50 μm apart. The probe was first lifted off the sample surface, then indented to a depth of 15 μm. A virtual contact point was determined^44^ to account for surface roughness. Hertz contact modulus, E*, was calculated at each point by fitting Hertz’ equation for spherical indentation of a half-space to the loading curve. We made no assumptions about the material’s Poisson’s ratio and therefore report Hertz contact modulus instead of Young’s Modulus. Indents exceeding 10 MPa were considered outside the soft tissue region of the growth plate and were discarded. Results were also confirmed using a nonlinear poroelasitc model, Hertz Biphasic Theory^45,46^.

### Macromer synthesis

A 10 kDa, 8-arm poly(ethylene glycol) (PEG) amine (JenKem) was modified with a norbornene group at the end of each arm^47^. Briefly, the PEG amine, dissolved in dimethylformamide (DMF) and dichloromethane (DCM), was reacted overnight, under argon, with eight times molar excess of 5-norbornene-2-carboxylic acid in the presence of three molar excess 1-[Bis(dimethylamino)methylene]-1H-1,2,3-triazolo[4,5-b]pyridinium 3-oxide hexafluorophosphate (HATU) and four molar excess N,N-diisopropylethylamine (DIPEA). The product was recovered and purified. Comparison of the area under the peak for the allylic hydrogen closest to the norbornene hydrocarbon (δ = 3.1–3.2 ppm) and the peak for the PEG backbone methyl group (δ = 3.4–3.85 ppm) in an ^1^H-NMR spectrum were used to determine a ~100% conjugation of norbornene to the 8-arm PEG. Thiolated chondroitin sulfate (ChS-SH) was then synthesized^3^. Briefly, Dimethyl 3,3′-dithioldipropinoate (DTP) was reacted with eight molar excess hydrazine hydrate. The product (DTP dihydrazide) was reacted with chondroitin sulfate A (Sigma Aldrich) in a two-molar excess at a pH of 4.75, followed by a reaction with 1-ethyl-3-(3-dimethylaminopropyl)carbodiimide. The reaction was stopped by adjusting the pH to 7.0. Dithiothreitol was added in a 6.5 molar excess and the pH was adjusted to 8.5. The product was recovered and purified. Comparison of the area under the peak for the methylene groups of DTP (δ = 2.5–2.6 and 2.6–2.8 ppm) and the area under the peak for the methyl group of the acetyl amine side chain of the chondroitin sulfate backbone (δ = 1.8–2.0 ppm) in an ^1^H-NMR spectrum were used to determine a ~18% conjugation of thiol groups per ChS molecule. For the cell-laden in vitro cell studies and the in vivo studies, TGFβ3 (Peprotech) was functionalized by addition of a thiol group by reaction with four molar excess Traut’s reagent in buffer, for one hour. Once functionalized, TGFβ3-SH was tethered to the PEGNB macromer via thiol:norbornene photoclick reaction.

### Composite formation

Poly(ethylene glycol) diacrylate (PEGDA 700, Aldrich) and pentaerythritol tetrakis(3-mercaptopropionate) (PETMP > 95%, Aldrich) were mixed at 99:1 wt% with 0.85 wt% diphenyl-(2,4,6-trimethylbenzoyl)phosphine oxide (TPO, 97%, Aldrich), as the photoinitiator, 0.05 wt% of 2,2′-Azobis(2-methylpropionitrile) (AIBN, 98%, Aldrich) as the thermal initiator and 0.8 wt% 2-(2-hydroxyphenyl)-benzotriazole derivative (Tinuvin1 CarboProtect1, BASF Company) as the photoabsorber. The photo-resin used for Formulation C differed in that 50 wt% water was added and the Tinuvin Caroboprotect photoabsorber was replaced for 0.08 wt% Quinoline Yellow (Aldrich) due to solubility. AIBN was added to the resin prior to performing the experiment to avoid degradation.

Three different formulations were used in this study to assess the impact of structural properties. All were created using CAD designs consisting of vertical 200 μm diameter pillars connected by a lattice on each side. Formulation A had 98 pillars, which yields a theoretical pillar volume ratio of 12.3% while Formulation B and C had 61 pillars corresponding to a theoretical 7.6% volume fraction of solid pillar structure in the CAD design. To connect these pillars Formulation A had a lattice with open regions of 256 μm^2^ and since the pillar array was less dense in Formulation B and C the open regions on the lattice in these designs were 256 × 167 μm^2^. Formulation A design was sliced into sixty 25 μm layers, Formulation B and C designs were sliced into thirty 50 μm layers, to form a structure with a height of 1.5 mm before swelling. Each layer was exposed for 6 s at an irradiation intensity of 14.4 mWcm^2^ as measured by a power meter at *λ* = 405 nm where the power was 0.9 +/− 0.1 mW for a 0.0025 cm^2^ square image (Model 2936-C, Newport). After printing, Formulation A structures were subsequently post-cured in an oven at a temperature of 105 +/− 5 °C under vacuum for 1 h. All were soaked in 100% ethanol for a minimum of 72 h.

### Cartilage-mimetic hydrogel

The biomimetic soft hydrogel was prepared by photopolymerization of 9 wt% poly(ethylene glycol) norbornene (PEGNB), 50 nM TGFβ3-SH, 1 wt% ChS-SH, 0.1 mM GCRGDS (GenScript), MMP-sensitive peptide (GCVPLS-LYSGCG, GenScript), and 0.05 wt% photoinitiator Irgacure 2959 (I2959) in phosphate-buffered saline (PBS) yielding a final thiol:ene ratio of 1:1. The hydrogel precursor solution was either polymerized directly or infilled into the 3D printed structure and then polymerized under 352 nm light for 8 min.

### 3D structure characterization

A 3D printed structure of a single pillar representing the polymerized resin material (*n* = 8) and the 3D print structure with the pillar array for each formulation both unfilled and filled (*n* = 5/group/condition) were assessed in unconfined compression (MTS Insight II; Eden Prairie, MN; 250 N load cell). The 3D printed structures were infilled with the cartilage-mimetic hydrogel precursor solution and then polymerized to create the composite (gel + 3D print) or were tested without the cartilage mimetic hydrogel infill (unfilled 3D print). All samples were swollen to equilibrium in PBS prior to testing. Following a 3 mN pre-load, a constant strain rate of 0.03 mm/s was applied to each sample. Single pillars were loaded until they failed while the 3D printed structures were strained to 12.5%. To account for the change in cross-sectional area with compression the modulus was derived from a fit of the linear region of the true stress-true strain curve (assuming a Poisson’s ratio of 0.5). For analysis of the composites and unfilled scaffolds, it is assumed that the contact force was evenly distributed throughout the contact surface. Cross-sectional area of the unfilled 3D prints was based on the theoretical pillar volume fraction. Atomic force microscopy (AFM, Cypher, Asylum Research) was used in fast force mapping (FFM) mode to measure the indentation modulus along the surface of a sectioned 3D printed structure and evaluate the efficacy of thermal post-curing to reduce material property fluctuations as a function of layer depth. A cryo-ultramicrotome (Leica, EM FC7) was used to generate a flat cross-section of the structure for accurate FFM. A silicone doped probe with a tip radius of curvature <10 nm (PPP-FMAuD-10 probe, Nanosensors, Switzerland) and a spring constant of 3.44 nN/nm was used as it is specifically tailored for FFM. To account for adhesion, AFM indentation modulus measurements were analyzed using a Johnson–Kendall–Roberts contact mechanics model^48^. Pillar diameter measurements were taken from SEM images. Images were scaled, and multiple measurements along the pillar height were taken and averaged for a single value of pillar diameter. A total of *n* = 13, 12, and 21 pillars were measured for Formulations A, B, and C, respectively.

### Fabrication of cell-laden hydrogels

Femoral bone marrow from New Zealand white rabbits was harvested and nucleated rMSCs were isolated using density gradient centrifugation^49^. At 60% confluency, cells were subsequently re-seeded at 2800 cells/cm^2^ and placed into a humidified 5% CO_2_, 37 °C incubator until passage 3. The in vitro study included two conditions, a cell-laden cartilage-mimetic hydrogel only (gel only) and the stiff 3D printed structure infilled with the cell-laden cartilage-mimetic hydrogel (gel + 3D print, Formulation A). Rabbit MSCs were encapsulated at 50 million cells mL^−1^ of filter-sterilized (0.22 µm) hydrogel precursor solution. For the gel only condition, the precursor solution was transferred to a 5 mm × 5 mm × 1 mm square Teflon mold and photopolymerized. For the gel + 3D print condition, the cell-laden precursor solution was pipetted into sterile 5 mm × 5 mm × 1 mm 3D printed structures and allowed to completely fill the interior. Both conditions were polymerized under 352 nm light at 5 mW cm^−2^ for 8 min. Cell-laden constructs were cultured for up to 9 weeks in chondrogenic differentiation medium (1% ITS + premix, 100 nM dexamethasone, 50 µg mL^−1^ L-ascorbic acid 2-phosphate, 50 U mL^−1^ penicillin, 50 mg mL^−1^ streptomycin, and 10 mg mL^−1^ gentamicin in high glucose Dulbecco’s modified Eagle media) under standard cell conditions of 37 °C with 5% CO_2_. Medium was exchanged every other day.

### Live cell imaging and histological analysis

Samples were taken from both conditions at nine weeks to visualize the encapsulated cells for assessment of viability and distribution within the hydrogel. Structures were incubated at 37 °C in a solution of 4 nM calcein AM (live cells) and 2 nM ethidium homodimer (dead cells). Stained samples were then immediately imaged on a confocal microscope (Ziess LSM 5 Pascal). At day 1 and at 3- and 9-weeks, samples were fixed in 4% paraformaldehyde. Half of the samples (*n* = 2 per time point) were embedded in glycol methacrylate (GMA) using the Technovit® 7100 kit (EMS) per manufacturer instructions. Samples were sectioned at 5 µm using a tungsten blade on an automated microtome. GMA-embedded sections were stained with hematoxylin and eosin (H&E) to visualize cell morphology and non-specific tissue deposition using light microscopy (Nikon Te-2000, Nikon Digital Sight DS-Qi1Mc). The remaining half (*n* = 2 per time point) were dehydrated and embedded in paraffin following standard protocols. Paraffin-embedded samples were sectioned to 5 µm. Sections were stained with Safranin-O/Fast Green to visualize sulfated glycosaminoglycans (sGAGs) using light microscopy (Zeiss Pascal, Olympus DP70). Immunohistochemistry was completed as follows. Deparaffinized and rehydrated samples were pretreated with specified enzymes (hyaluronidase 200 U mL^−1^ for aggrecan and collagen II, chondroitinase ABC and keratinase I (4 mU) for aggrecan, pepsin (280 kU) for collagen X) for one hour at 37 °C and antigen retrieval (collagen X and aggrecan). Sections were blocked with 10% Normal Goat Serum (NGS) and 2% Bovine Serum Albumin (BSA) in Tris Buffered Saline (TBS) (PEG, collagen II, and collagen X) or 1% BSA in PBS (aggrecan) and permeabilized with 1% BSA and 0.25% Triton-X 100 in TBS (collagen II and collagen X) or 1% BSA and 0.25% Triton-X 100 in PBS (aggrecan). Sections were subsequently treated with primary antibodies against aggrecan (1:15, Abcam ab3778), collagen type II (1:100, Iowa hybridoma bank), collagen X (1:200, Abcam ab49945), and PEG (1:50 Academia Sinica Anti-PEG 6.3) followed by secondary antibodies with conjugated AlexaFluor 488 or 546 probes and counterstained with DAPI for nucleus detection. Negative controls were prepared by omitting the primary antibody. A laser scanning confocal microscope (Nikon Eclipse Ti, Nikon A1R) was used to acquire images at ×40 magnification (Plan Fluor 40X oil objective). Representative images were selected from those acquired.

### Analysis of total MMPs in media

Total MMP in media was measured with the Anaspec Generic MMP Assay kit (AS-71158) following manufacturer instructions (*n* = 9). Briefly, 8 mM APMA enzyme activating solution was added to each of the media samples to activate MMPs and incubated for one hour at 37 °C. MMP substrate solution was then added to activated samples and incubated for one hour and fluorescence (490/520 nm) was measured. Total MMP in solution was calculated using collagenase type-II as a standard. Measurements for each week, within a given sample, were combined to yield the total MMPs produced during the duration of the study.

### Animal preparation

All procedures complied with the Guide for the Care and Use of Laboratory Animals and were approved by the Institutional Animal Care and Use Committee at the University of Colorado Denver. The first in vivo study was used to evaluate the impact of treatment modality. Thirty-nine 6-week-old New Zealand white rabbits (*n* = 24 males, *n* = 15 females) were assigned to one of four treatment groups: (1) untreated (*n* = 10), (2) autologous fat graft (*n* = 9), (3) cartilage-mimetic hydrogel (gel only) (*n* = 10), and (4) 3D printed growth plate mimetic composite (gel + 3D print) (*n* = 10, Formulation A). To evaluate the effect of scaffold design, a second in vivo study was performed using twenty-four 6-week-old New Zealand white rabbits (*n* = 12 males, *n* = 12 females) equally assigned to one of three gel + 3D print formulation groups: Formulation A (*n* = 8), Formulation B (*n* = 8), and Formulation C (*n* = 8). Right limbs were injured, and the left limbs served as a non-operative control.

### Surgical procedures

An initial 5 × 5 × 1 mm proximal tibial physeal injury was created from an anterior-medial approach using a 1 mm drill bit and intraoperative radiographic images for defect positioning^33^. A second surgical procedure was performed after 3 weeks on all animals. During this second surgery, the same approach was used, callus tissue was removed and a 2 mm drill bit was used to create a 6 × 6 × 2 mm resection of the subsequent bony bar formation. The resected tissue area was then filled with either a fat graft (taken from inguinal area), gel only, or a gel + 3D print condition. For these studies, a lithium acylphosphonate (LAP) photoinitiator was used in the hydrogel precursor solution and a handheld 405 nm light was applied for 1 min. In the gel + 3D print condition, the 3D print was infilled with the hydrogel precursor solution and placed in the defect. An additional amount of precursor solution was added to ensure complete infilling, followed by light polymerization. All animals were allowed normal cage activity, exercise, and food and water at-lib. Animals were euthanized via overdose of isoflurane and intracardiac injection of pentobarbital (150 mg/kg) at 8 weeks post treatment for the first in vivo study evaluating different treatments and at 12 weeks post treatment for the second in vivo study evaluating scaffold formulation.

### X-ray image processing

Anteroposterior radiographic images using 50 kV and 0.4 mA (AJEX Meditech Ltd; J type stand unit) were taken of the lower limbs before growth plate injury, during treatment, and at 4- and 8-weeks post treatment for the first study, and at 5-, 8-, and 12-weeks post treatment for the second study. These images were used to measure tibial length and tibial angle of both the injured and control limbs with ImageJ (v 1.53c with Java 1.8.0_172). Tibial length was measured as the distance between the tibial distal growth plate and the proximal tibial plateau at 50% of the full width. Tibial angle was assessed as the angle between the leg length measure and the average angle across the entire plateau. Tibial length change was assessed as change from time of treatment.

### MicroCT bone analysis

Following euthanasia, proximal tibias from treatment limbs were harvested, wrapped in parafilm to prevent dehydration, and imaged. Animals in the first study were imaged with an Xradia 520 Versa (Carl Zeiss XRM, Pleasanton, CA, USA) at a voxel size of 26 μm (80 kV, 86–88 μA, 0.5 s excitation, 801 projections). Automated centering and beam hardening corrections were applied using the Scout-and-Scan Control System Reconstructor software (version 14.0.14829.38124). Animals from the second study were imaged with SCANCO μCT50 (Scanco Medical, Basserdorf, Switzerland) at a voxel size of 17.2 μm (70 kV, 200 μA, 0.5 s excitation, 1000 projections). Reconstructions were imported into Dragonfly (version 4.1, Object Research Systems, Montreal, Canada) and an Otsu’s algorithm^50^ was implemented to separate ranges of the histogram and binarize the data set. Segmentation of cortical and trabecular bone was performed using the Buie Method^51^, excluding the fibula, and evaluating equal volumes above and below the growth plate to accommodate any variability in scaffold position. The bone analysis plugin for Dragonfly (version 4.1, Object Research Systems, Montreal, Canada) was used to evaluate bone volume fraction (BV/TV) as well as trabecular thickness (Tb.Th.) and trabecular spacing (Tb.Sp.) using a sphere fitting algorithm^52^.

### Histology and immunohistochemistry evaluation of in vivo study samples

Tissues were fixed in 10% neutral-buffered formalin for 7 days and then decalcified in 14% ethylenediaminetetraacetic acid for up to 4 months. After decalcification, samples were embedded in paraffin, coronally sectioned (5 μm), standard Alcian Blue Hematoxylin was performed to assess repair tissue. For immunohistochemistry, samples were pretreated (hyaluronidase 200 U mL^−1^) for one hour at 37 °C. Sections were blocked with 10% Normal Goat Serum in Tris Buffered Saline (TBS) and subsequently treated with a primary antibody against collagen type II (1:100, Iowa hybridoma bank) overnight at 4 °C. The secondary antibody, peroxidase-conjugated goat anti-mouse IgG (Jackson ImmunoResearch), was diluted 1:500 and incubated at room temperature for 1 h. Slides were developed using the DAB substrate kit (Vector Laboratories). Negative controls were prepared by omitting the primary antibody. Slides were digitized with the Axio Scan.Z1 (Zeiss).

### Statistical analysis

All data reported are mean ± standard deviation. Pillar diameter measures were not homogeneous or normally distributed so a Kruskal–Wallis test (one-sided) was used to evaluate differences across formulations. Compressive modulus across the three formulations was evaluated using a one-way analysis of variance (ANOVA) with Tukey’s HSD post hoc test to correct for multiple comparisons (two-sided). Differences between empty and infilled scaffolds were assessed with a *t*-test (two-sided). A two-sample *t*-test was used to assess differences between the gel only and gel + 3D print for MMPs (two-sided). Comparisons between groups in all in vivo analyses were evaluated using a two-way ANOVA with Tukey’s HSD post hoc test for multiple comparisons (two-sided). All statistical tests were evaluated as two-sided, and significance was set at *p* < 0.05. All statistical analysis was performed using GraphPad Prism (v 9.0.1, GraphPad Software).

### Reporting summary

Further information on research design is available in the Nature Research Reporting Summary linked to this article.

## Supplementary information

REPORTING SUMMARY

## Data Availability

The data included in this manuscript are available from the corresponding author upon request.
