# Supplementary material for: A 3D printed mimetic composite for the treatment of growth plate injuries in a rabbit model
Source: NPJ Regen Med. 2022 Oct 19;7:60. doi: 10.1038/s41536-022-00256-1 (PMC9581903; doi:10.1038/s41536-022-00256-1)
Supplement: Supplementary file 1 — REPORTING SUMMARY [file 41536_2022_256_MOESM1_ESM.pdf]

nature portfolio

Corresponding author(s): Karin A. Payne, PhD

Last updated by author(s): Sep 13, 2022

Reporting Summary

Nature Portfolio wishes to improve the reproducibility of the work that we publish. This form provides structure for consistency and transparency in reporting. For further information on Nature Portfolio policies, see our Editorial Policies and the Editorial Policy Checklist.

Statistics

For all statistical analyses, confirm that the following items are present in the figure legend, table legend, main text, or Methods section.

n/a	Confirmed	
☐	☒	The exact sample size (n) for each experimental group/condition, given as a discrete number and unit of measurement
☐	☒	A statement on whether measurements were taken from distinct samples or whether the same sample was measured repeatedly
☐	☒	The statistical test(s) used AND whether they are one- or two-sided<br>*Only common tests should be described solely by name; describe more complex techniques in the Methods section.*
☒	☐	A description of all covariates tested
☐	☒	A description of any assumptions or corrections, such as tests of normality and adjustment for multiple comparisons
☐	☒	A full description of the statistical parameters including central tendency (e.g. means) or other basic estimates (e.g. regression coefficient) AND variation (e.g. standard deviation) or associated estimates of uncertainty (e.g. confidence intervals)
☐	☒	For null hypothesis testing, the test statistic (e.g. F, t, r) with confidence intervals, effect sizes, degrees of freedom and P value noted<br>*Give P values as exact values whenever suitable.*
☒	☐	For Bayesian analysis, information on the choice of priors and Markov chain Monte Carlo settings
☒	☐	For hierarchical and complex designs, identification of the appropriate level for tests and full reporting of outcomes
☒	☐	Estimates of effect sizes (e.g. Cohen's d, Pearson's r), indicating how they were calculated

Our web collection on statistics for biologists contains articles on many of the points above.

Software and code

Policy information about availability of computer code

Data collection	AFM- Cypher, Asylum Research<br>microindenation - Hysitron TI 950 TriboIndenter<br>gross mechanical testing - MTS Insight II; Eden Prairie, MN; 250 N load cell<br>in vitro imaging - Nikon Eclipse Ti, Nikon A1R; Zeiss Pascal, Olympus DP70; Ziess LSM 5 Pascal<br>xray - (AJEX Meditech Ltd; J type stand unit)<br>microCT - Xradia 520 Versa (Carl Zeiss XRM, Pleasanton, CA, USA) and SCANCO µCT50 (Scanco Medical, Basserdorf, Switzerland)<br>in vivo Histology images - Axio Scan.Z1 (Zeiss)
Data analysis	mechanical testing - MATLAB (Mathworks, Natick, MA, R2019b)<br>xray - ImageJ (v 1.53c with Java 1.8.0_172)<br>microCT - Dragonfly (version 4.1, Object Research Systems, Montreal, Canada)<br>in vivo Histology images - ImageJ (v 1.53c with Java 1.8.0_172)<br>Statistical analysis - GraphPad Prism (v 9.0.1, GraphPad Software)<br>Other - Adobe illustrator (2022 26.2.1)

For manuscripts utilizing custom algorithms or software that are central to the research but not yet described in published literature, software must be made available to editors and reviewers. We strongly encourage code deposition in a community repository (e.g. GitHub). See the Nature Portfolio guidelines for submitting code & software for further information.

Data

Policy information about availability of data

All manuscripts must include a data availability statement. This statement should provide the following information, where applicable:

- Accession codes, unique identifiers, or web links for publicly available datasets
- A description of any restrictions on data availability
- For clinical datasets or third party data, please ensure that the statement adheres to our policy

The data included in this manuscript are available from the corresponding author upon request.

Field-specific reporting

Please select the one below that is the best fit for your research. If you are not sure, read the appropriate sections before making your selection.

☒ Life sciences ☐ Behavioural & social sciences ☐ Ecological, evolutionary & environmental sciences

For a reference copy of the document with all sections, see nature.com/documents/nr-reporting-summary-flat.pdf

Life sciences study design

All studies must disclose on these points even when the disclosure is negative.

Sample size	The determination of treatment group sample size is based on data our lab previously generated on growth plate injuries in rabbits. Outcome measures include bony repair tissue by microCT, varus angulation (degrees), and bone length (cm). Taking the means and standard deviations from our data, we determined group sample size using SISA. With an alpha of 5% and power of 95%.
Data exclusions	Indents exceeding 10 MPa were considered outside the soft tissue region of the growth plate and were discarded.
Replication	n/a
Randomization	Scaffolds and hydrogels were specifically designed and fabricated for their respective formulations, and thus no randomization was employed. Animals were randomly assigned to treatment groups with roughly equal distributions of males and females being placed in each group. However, due to lack of differences, sex was not ultimately evaluated as a covariate.
Blinding	Where possible samples were denoted as numbers rather than identified by group/treatment name.

Reporting for specific materials, systems and methods

We require information from authors about some types of materials, experimental systems and methods used in many studies. Here, indicate whether each material, system or method listed is relevant to your study. If you are not sure if a list item applies to your research, read the appropriate section before selecting a response.

Materials & experimental systems

n/a	Involved in the study	
☐	☒	Antibodies
☒	☐	Eukaryotic cell lines
☒	☐	Palaeontology and archaeology
☐	☒	Animals and other organisms
☒	☐	Human research participants
☒	☐	Clinical data
☒	☐	Dual use research of concern

Methods

n/a	Involved in the study	
☒	☐	ChIP-seq
☒	☐	Flow cytometry
☒	☐	MRI-based neuroimaging

Antibodies

Antibodies used	Primary: aggrecan (Abcam, ab3778), collagen type II (Iowa hybridoma bank, CIIC1), collagen x (Abcam, ab49945), PEG (Academia Sinica , 6.3-PABG-A)<br>Secondary: peroxidase conjugated goat anti-mouse IgG (Jackson ImmunoResearch 115-035-166)<br>Goat anti-mouse IgG (H+L) Highly Cross-Absorbed secondary Antibody, Alexa Fluor 546 (Invitrogen, #A11030)<br>Goat anti-mouse IgG (H+L) Cross-Absorbed secondary Antibody, Alexa Fluor 488 (Invitrogen #A-11001)
Validation	Each antibody was validated according to the manufacturer's protocols.

Animals and other organisms

Policy information about studies involving animals; ARRIVE guidelines recommended for reporting animal research

Laboratory animals	New Zealand White Rabbits, mix of male and female (n=36 and n=27 respectively), initial surgery at 6 weeks old
Wild animals	No wild animals were used
Field-collected samples	No field-collected samples were used
Ethics oversight	All procedures complied with the Guide for the Care and Use of Laboratory Animals and were approved by the Institutional Animal Care and Use Committee at the University of Colorado Denver.

Note that full information on the approval of the study protocol must also be provided in the manuscript.
